# Vasculogenically conditioned peripheral blood mononuclear cells inhibit mouse immune response to induced pluripotent stem cell-derived allogeneic cardiac grafts

**DOI:** 10.1371/journal.pone.0217076

**Published:** 2019-05-28

**Authors:** Noriyuki Kashiyama, Shigeru Miyagawa, Satsuki Fukushima, Takuji Kawamura, Ai Kawamura, Shohei Yoshida, Yuki Nakamura, Akima Harada, Haruchika Masuda, Koichi Toda, Takayuki Asahara, Yoshiki Sawa

**Affiliations:** 1 Department of Cardiovascular Surgery, Osaka University Graduate School of Medicine, Suita City, Osaka, Japan; 2 Depertment of Regenerative Medicine, Tokai University School of Medicine, Isehara City, Kanagawa, Japan; University of Alabama at Birmingham, UNITED STATES

## Abstract

Allogeneic transplantation of induced pluripotent stem cell (iPSC)-derived cardiomyocytes is apromising treatment for cardiac diseases, although immune rejection by the recipient poses a concern. In this study, we aimed to investigate whether concomitant transplantation of vasculogenically conditioned peripheral blood mononuclear cells, which are otherwise immunosuppressive, may enhance graft survival. Luciferase-transduced, iPSC-derived cardiomyocytes from C57BL/6 mice were transplanted to the dorsal subcutaneous space of syngeneic C57BL/6 mice (n = 19), allogeneic Balb/c mice treated with (n = 20) or without (n = 20) immunosuppressants, and those injected with vasculogenically conditioned peripheral blood mononuclear cells (n = 20). Although graft survival, assessed by bioluminescence, was comparable among the groups initially, it improved significantly at days 7 and 10 in allogeneic transplanted mice treated with vasculogenically conditioned peripheral blood mononuclear cells than in others (*P* < 0.01). Our results proved that cell-based immunosuppression may boost clinical outcomes from allogeneic cell therapy.

## Introduction

Regenerative therapy based on induced pluripotent stem cells (iPSC) is already available in the clinic [1]. In addition, the safety and efficacy of iPSC-derived cardiomyocytes as treatment for ischemic heart failure have now been demonstrated in some large animals [2, 3]. However, pre-made allogeneic iPSCs are probably more feasible to use in the clinic, despite likely immune rejection by the host, since autologous iPSCs are time-consuming and expensive to establish for each patient [4]. Accordingly, intensive immunosuppressive therapy is required to ensure graft survival from natural killer cells or immune reactions against minor antigens, even though MHC homo-to-hetero transplantation may also mitigate host immunity against iPSC-derived grafts [5–9]. Unfortunately, immunosuppressants have severe side effects. Hence, immunosuppressive cells such as mesenchymal stem cells have been investigated as alternatives [10, 11], although these would have to be obtained by invasive bone marrow aspiration. Intriguingly, vasculogenically conditioned peripheral blood mononuclear cells, which are strongly vasculogenic and were originally established as regenerative therapy for ischemic disease [12, 13], were found to also contain immunosuppressive cells such as regulatory T cells or M2 macrophages [15]. As these cells can be simply generated from peripheral blood, we have now tested the possibility that concomitant transplantation of such cells may enhance survival of allogeneically grafted iPSC-derived cardiomyocytes by suppressing host immunity.

## Materials and methods

Animal care was compliant with the Guide for Care and Use of Laboratory Animals by the National Institutes of Health. Protocols were approved by the Ethics Review Committee for Animal Experimentation at Osaka University Graduate School of Medicine (reference number 25-025-051).

### Differentiation of murine iPSCs into cardiac sheets

**Fig 1:**
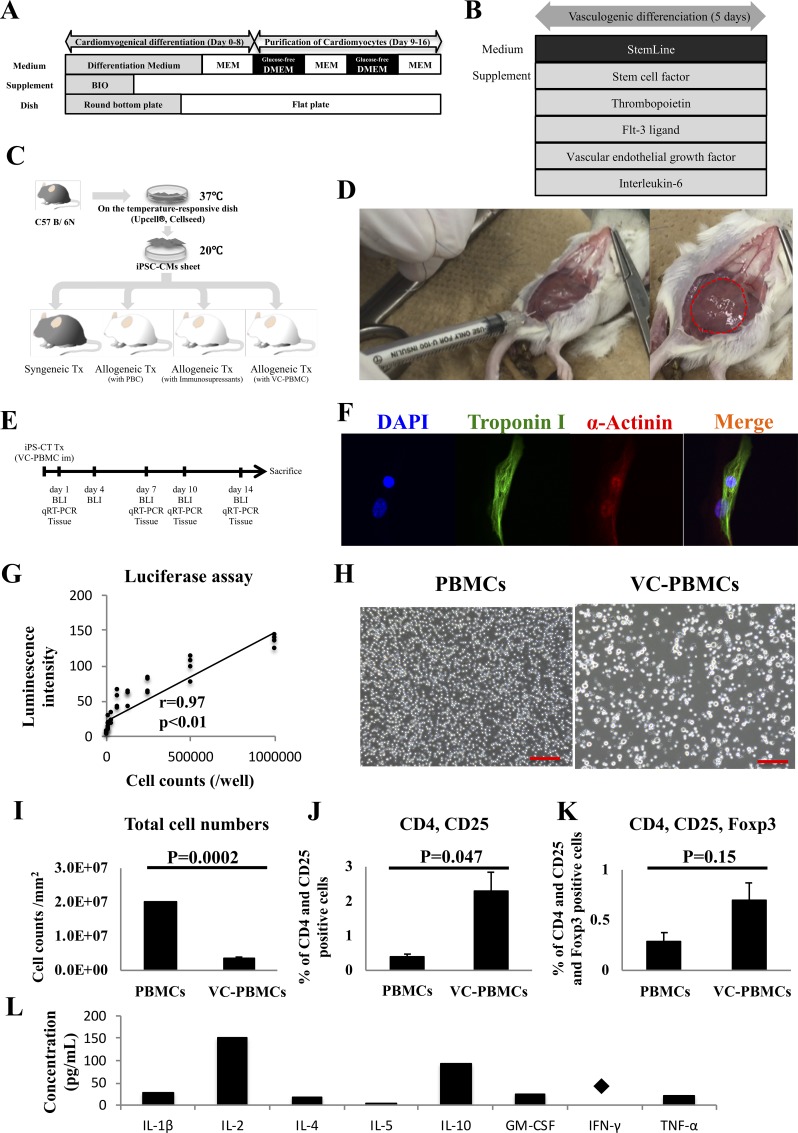
Experimental protocols. **A**, Differentiation and purification of cardiomyocytes from murine iPSCs. **B**, Vasculogenic conditioning of peripheral blood mononuclear cells. **C**, Protocol to inject vasculogenically conditioned peripheral blood mononuclear cells and transplant iPSC-derived cardiac sheets. **D**, Transplantation of cardiac sheets derived from C57BL/6 iPSCs into the subcutaneous space of syngeneic C57BL/6 mice and allogeneic Balb/c mice, with or without treatment with immunosuppressants or coinjection of vasculogenically conditioned peripheral blood mononuclear cells. Dotted lines indicate iPSC-derived cardiac sheets. **E**, Bioluminescence imaging and tissue analysis by staining or RT-PCR. **F**, Confocal laser scanning micrographs of iPSC-derived cardiac sheets stained with DAPI and fluorescently labeled antibodies to α-actinin (Alexa Fluor 647) and troponin I (Alexa Fluor 488). **G**, Luminescence intensity as a function of number of iPSC-derived cardiomyocytes. **H**, Micrographs of unconditioned and vasculogenically conditioned peripheral blood mononuclear cells. Scale bar, 100 μm. **I-K**, Total cell number and frequency of CD4^+^CD25^+^ and CD4^+^CD25^+^Foxp3^+^ cells before and after vasculogenic conditioning of peripheral blood mononuclear cells. **L**, Secretion of cytokines and growth factors *in vitro*. Several factors potentially involved in immune reactions were detected at relatively high concentrations in the medium. *, *P* < 0.05; N.S., not significant.

As described previously [15], luciferase was transduced into the 959A2-1 murine iPSCs, which was generated from C57BL/6 (B6) mouse embryonic fibroblasts by introducing Yamanaka factors such as Oct3/4, Sox2, Klf4, and c-Myc without viral vectors. These iPSCs were cultured without serum or feeder cells in ESGRO Complete PLUS Clonal Grade Medium (Millipore, Waltham, MA), differentiated into cardiomyocytes as described previously, and purified on glucose-free medium supplemented with lactic acid [16] (Fig 1A).

### Vasculogenic conditioning of murine peripheral mononuclear cells

As described previously [14], peripheral blood mononuclear cells from Balb/c mice were vasculogenically conditioned for five days in StemLine II medium (Sigma Aldrich) containing stem cell factor, thrombopoietin, Flt-3 ligand, vascular endothelial growth factor, and interleukin-6, all of which were obtained from PeproTech (Rocky Hill, NJ) (Fig 1B).

### Cotransplantation

Cardiac tissue (5 × 10^6^ cells) derived from luciferase-transduced iPSCs obtained from an adult C57BL/6 male mouse (10 weeks old, 20–25 g, CLEA, Tokyo, Japan) were transplanted into the dorsal subcutaneous space of syngeneic C57BL/6 mice (n = 19), allogeneic Balb/c mice treated with (n = 20) or without (n = 26) immunosuppressants, and allogeneic Balb/c mice that also received a subfascial injection of 5 × 10^5^ vasculogenically conditioned peripheral blood mononuclear cells (n = 26) (Fig 1C and 1E). The immunosuppressant tacrolimus was administered to the corresponding treatment group by continuous infusion from an ALZET osmotic pump (DURECT Corp., Cupertino, CA) immediately prior to transplantation. The drug was delivered at 0.5 mg/kg/day into the peritoneal space to maintain serum trough levels > 10 ng/mL until the end of the study. All surgeries and sacrifices were performed under deep anesthesia with isoflurane enough to minimize the animal suffering.

### *In vitro* T cell suppression assay

To assess the suppressive activity of vasculogenically conditioned peripheral blood mononuclear cells against T cells, splenic mononuclear cells (2.5 Å) were isolated from Balb/c mice, and incubated at 10^5^ cells/well on plates coated with (BIOCOAT 96-well anti-mouse CD3 T cell activation plate; BD) or without anti-mouse CD3 and filled with 200 μL RPMI 1640 (Sigma-Aldrich) containing 10% fetal bovine serum. Coated plates were also supplemented with 2 μg/mL anti-mouse CD28 (BD bioscience). After 48 hours, cytokines in culture supernatants were quantified with the Bio-Plex Pro^TM^ Mouse Cytokine 8-plex system (BIO-RAD). Wells were also reacted with 20 μL Cell Counting Reagent (Dojindo, Kumamoto, Japan), and the absorbance at 450 nm was measured after 2 hours. The stimulation index was calculated by dividing the absorbance from CD3/CD28 co-stimulated cells by the absorbance from unstimulated cells. Cytokines released by vasculogenically conditioned peripheral blood mononuclear cells only were also quantified in culture supernatants using the Bio-Plex Pro^TM^ Mouse Cytokine 8-plex system (BIO-RAD).

### Flow cytometry

Unconditioned or vasculogenically conditioned peripheral blood mononuclear cells were incubated for 12 hours at 37°C and 1 × 10^6^ cells/mL in RPMI 1640 medium containing 10% FBS, 25 ng/mL phorbol-12-myristate-13-acetate (Promega, Madison, WI), and 11 g/mL ionomycin (Wako Pure Chemical Industries, Ltd., Osaka, Japan). In the last 3 hours of incubation, cells were also treated with 21 mol/L monensin (BioLegend, San Diego, CA). Subsequently, cells were washed, harvested in PBS with 2 mmol/L EDTA, stained with CD4 and CD25 antibodies conjugated to PerCP/Cy5.5 and CD25, washed, resuspended in PBS with 2 mmol/L EDTA, aliquoted, and fixed with Foxp3 Fix/Perm Buffer Set (BioLegend) for intranuclear staining with anti-Foxp3 conjugated to fluorescein isothiocyanate. CD4^+^CD25^+^ helper T cells and CD4^+^CD25^+^Foxp3^+^ regulatory T cells were quantified on an LSRFortessa cell analyzer (BD Biosciences) with in FlowJo software (Tree Star, Ashland, OR), and are reported as % of total lymphocytes. The total number of unconditioned or vasculogenically conditioned cells was also determined.

### Bioluminescence imaging

Graft survival was monitored at day 1, 4, 7, 10, and 14 by bioluminescence imaging on an IVIS Lumina II (Perkin Elmer, Waltham, MA). In brief, Rediject D-Luciferin Ultra Bioluminescence Substrate (Perkin Elmer) was administered intraperitoneally at 150 mg/kg body weight 6 minutes prior to imaging. Animals were then placed in a light-tight chamber, and photons emitted from luciferase-expressing cells were collected for five minutes per image. Images were analyzed in Living Image Software (PerkinElmer). To verify the correlation between luminescence intensity and number of iPSC-derived cardiomyocytes, cells were incubated for 24 hours in 96-well plates containing DMEM and 15% FBS, and assayed using a luciferase assay system (Promega) on a Synergy HT microplate reader equipped with an injector and with time-resolved fluorescence capability (BioTek).

### Histology and immunohistolabeling

Dissociated iPSC-derived cardiomyocytes were cultured on 4-well Lab-TekII chamber slides (Thermo Scientific), fixed with 4% paraformaldehyde, probed with rabbit antibodies to α-actinin (Sigma-Aldrich) and troponin I (Abcam, Cambridge, United Kingdom), and visualized with donkey anti-rabbit IgG conjugated to Alexa Fluor 647 and goat anti-rabbit IgG conjugated to Alexa Fluor 488 (Invitrogen). Nuclei were stained with 4',6-diamidino-2-phenylindole dihydrochloride (DAPI), and specimens were imaged on an FV1200 confocal laser scanning microscope (Olympus, Tokyo, Japan). Heart tissues were collected at day 1, 7, 10, and 14 after transplantation, fixed with 10% buffered formalin, embedded in paraffin, serially sectioned at 5 μm, deparaffinized in xylene, dehydrated in graded ethanol, and autoclaved in 0.01 M citrate buffer to expose antigens. Sections were then immersed in methanol containing 3% hydrogen peroxide, probed with rabbit antibodies to CD3 (clone SP7, ab116669, Abcam) and Foxp3, labeled with biotinylated anti-rabbit IgG (DAKO, Glostrup, Denmark) and peroxidase-conjugated streptavidin (GE Healthcare, Little Chalfont, United Kingdom), stained with biphenyl-3,3',4,4'-tetramin (Wako Pure Chemical), and imaged on a Biorevo BZ-9000 (Keyence, Osaka, Japan). The number of CD3 and Foxp3 positive cells were counted in 3 to 4 high-power fields (HPFs) per slide using Image J (National Institutes of Health, Bethesda, Maryland).

### Reverse-transcription and quantitative polymerase chain reaction

Total RNA was extracted from subcutaneous tissue using RNeasy RNA Fibrous Tissue Mini Kit (Qiagen, Hilden, Germany), and reverse transcribed into cDNA using TaqMan reverse transcription reagents (Applied Biosystems). CD247 (Mm00446171_m1), FOXP3 (Mm00475162_m1), IL-10 (Mm00439614_m1), IL-1β (Mm00434228_m1), CD68 (Mm03047343_m1), arginase-1 (Mm00475988_m1), mannose receptor (Mm01329362_m1), NOS2 (Mm00440502_m1), CCR2 (Mm99999051_gH), IL-2 (Mm00434256_m1), IFN-γ (Mm01168134_m1), and TNF-α (Mm00443258_m1) were quantified by quantitative real-time polymerase chain reaction on an ABI PRISM 7700 system (Applied Biosystems), and normalized to GAPDH (Mm99999915_g1) in each sample in duplicate experiments. Relative expression was then calculated and compared among groups.

### Statistical analysis

Continuous variables are reported as mean ± standard deviation, and were compared by Wilcoxon signed-rank test in JMP 13.0.0 (SAS Institute, Cary, NC). Correlations between continuous variables were tested with Pearson correlation coefficient (r). A probability value < 0.05 was considered statistically significant.

## Results

### Generation of iPSC-derived cardiomyocytes with transduction of luciferase gene

Cardiomyocytes were successfully differentiated from luciferase-transduced murine iPSCs using a well-established protocol (Fig 1A). These cardiomyocytes were spindle-shaped, and contained well-aligned sarcomeres in the cytoplasm, as assessed by immunohistological labeling of α-actinin and troponin I (Fig 1F). Luminescence intensity was strongly correlated with cell count (r = 0.97, *P* < 0.01), suggesting that luminescence can be used to evaluate graft survival quantitatively (Fig 1G).

### Regulatory T cells were included in vasculogenic conditioning of peripheral blood mononuclear cells with immunosuppressive cytokines

Murine peripheral blood mononuclear cells were vasculogenically conditioned using cytokine cocktails as previously described (Fig 1B). Based on microscopy, large cells were proportionally more common in conditioned cells than in unconditioned cells (Fig 1H). However, the increase in the number of vasculogenically conditioned cells was modest at 0.18 ± 0.015-fold per well (*P* < 0.01, Fig 1I), while CD4^+^CD25^+^ helper T cells significantly increased from 0.42 ± 0.06 to 2.30 ± 0.55% of total cells (*P* = 0.047), while CD4^+^CD25^+^Foxp3^+^ regulatory T cells accumulated only slightly from 0.28 ± 0.09 to 0.70 ± 0.18% (*P* = 0.15) (Fig 1J and 1K). Finally, vasculogenically conditioned cells (3.2 × 10^6^ cells/mL) abundantly secreted IL-2 (150.0 pg/mL) and IL-10 (92.3 pg/mL), in comparison to IL-1β (27.4 pg/mL), IL-4 (18.2 pg/mL), IL-5 (3.9 pg/mL), GM-CSF (23.2 pg/mL), and TNF-α (20.83 pg/mL). IFN-γ was not detected (Fig 1L).

### Graft survival was prolonged in allogeneic transplantation with vasculogenically conditioned peripheral blood mononuclear cells

**Fig 2:**
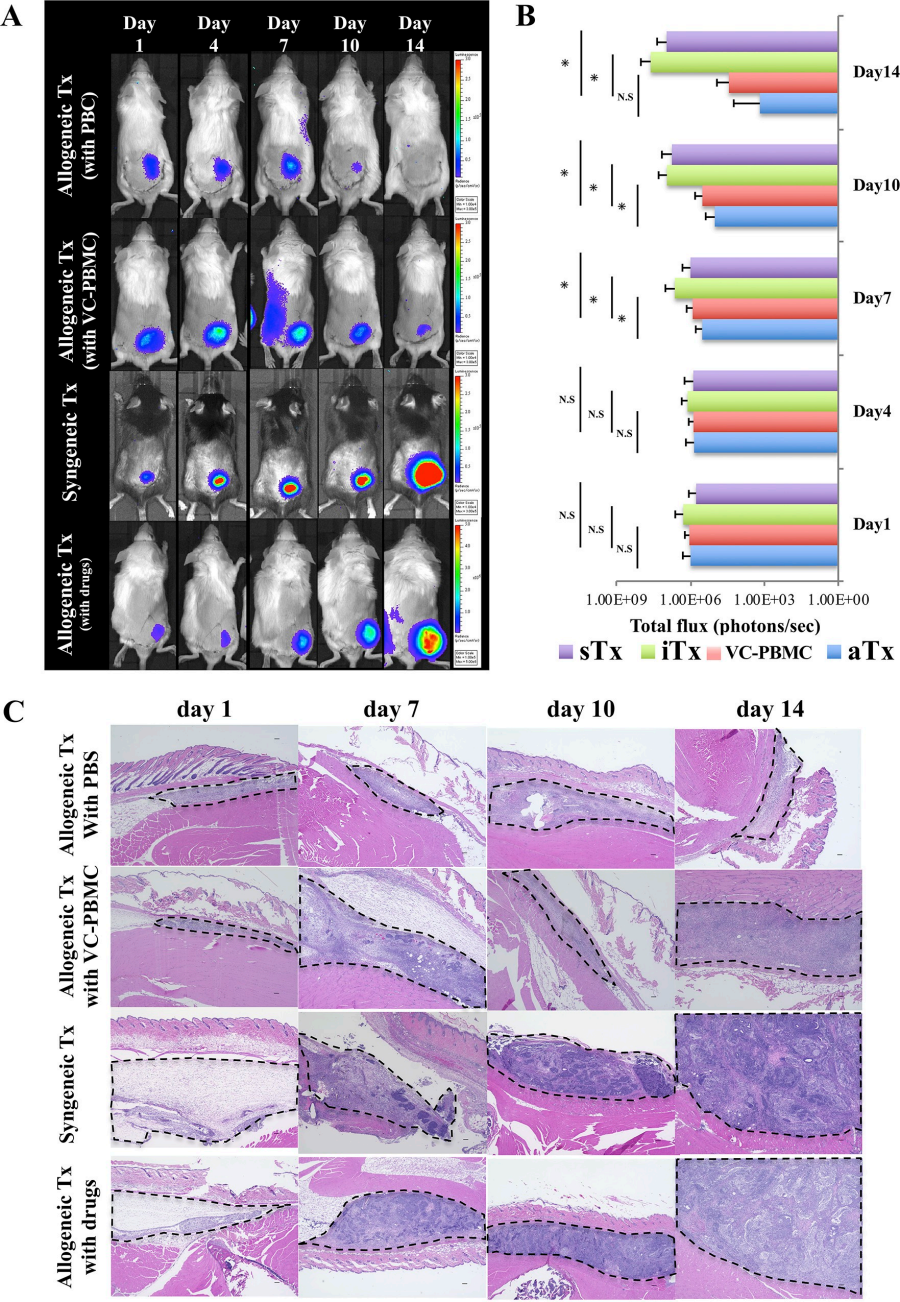
Bioluminescence imaging and hematoxylin and eosin staining of subcutaneous tissue. **A**, Time course of graft survival as assessed by bioluminescence imaging. **B**, Quantification of bioluminescence. **C**, Hematoxylin and eosin staining of serially sectioned subcutaneous tissues collected at days 1, 7, 10, and 14. Dotted lines indicate surviving iPSC-derived cardiac sheets. Scale bar, 100 μm. *, *P* < 0.05; N.S., not significant.

Grafted luciferase-expressing cardiomyocytes, as assessed by bioluminescence, diminished over 14 days in allogeneically transplanted mice that did or did not receive vasculogenically conditioned peripheral blood mononuclear cells, but increased in syngeneically transplanted mice, as well as in allogeneically transplanted mice treated with immunosuppressants, suggesting teratoma formation. On day 4, bioluminescence was comparable among all groups at 7.53 ± 9.58 × 10^5^ photons/sec in syngeneically transplanted mice, 7.38 ± 4.16 × 10^5^ photons/sec and 7.01 ± 8.05 × 10^5^ photons/sec in allogeneically transplanted mice injected with and without conditioned cells, and 1.29 ± 0.94 × 10^6^ photons/sec in allogeneically transplanted mice treated with immunosuppressants (*P* = 0.1). However, bioluminescence at subsequent time points was significantly higher in allogeneically transplanted mice treated with immunosuppressants than in the same mice treated with or without conditioned cells (*P* < 0.01 and *P* < 0. 001 at day 7, *P* < 0.01 and *P* < 0.001 at day 10, and P = 0.01 and P = 0.001 at day 14). On the other hand, bioluminescence was comparable at day 1 and 4 between allogeneically transplanted mice injected with and without conditioned cells, but was higher in the former at day 7 and 10 before returning to comparable levels at the end of the experiment (Fig 2A and 2B).

### Activated T cells were histologically infiltrated into the transplanted site of allogeneic graft

**Fig 3:**
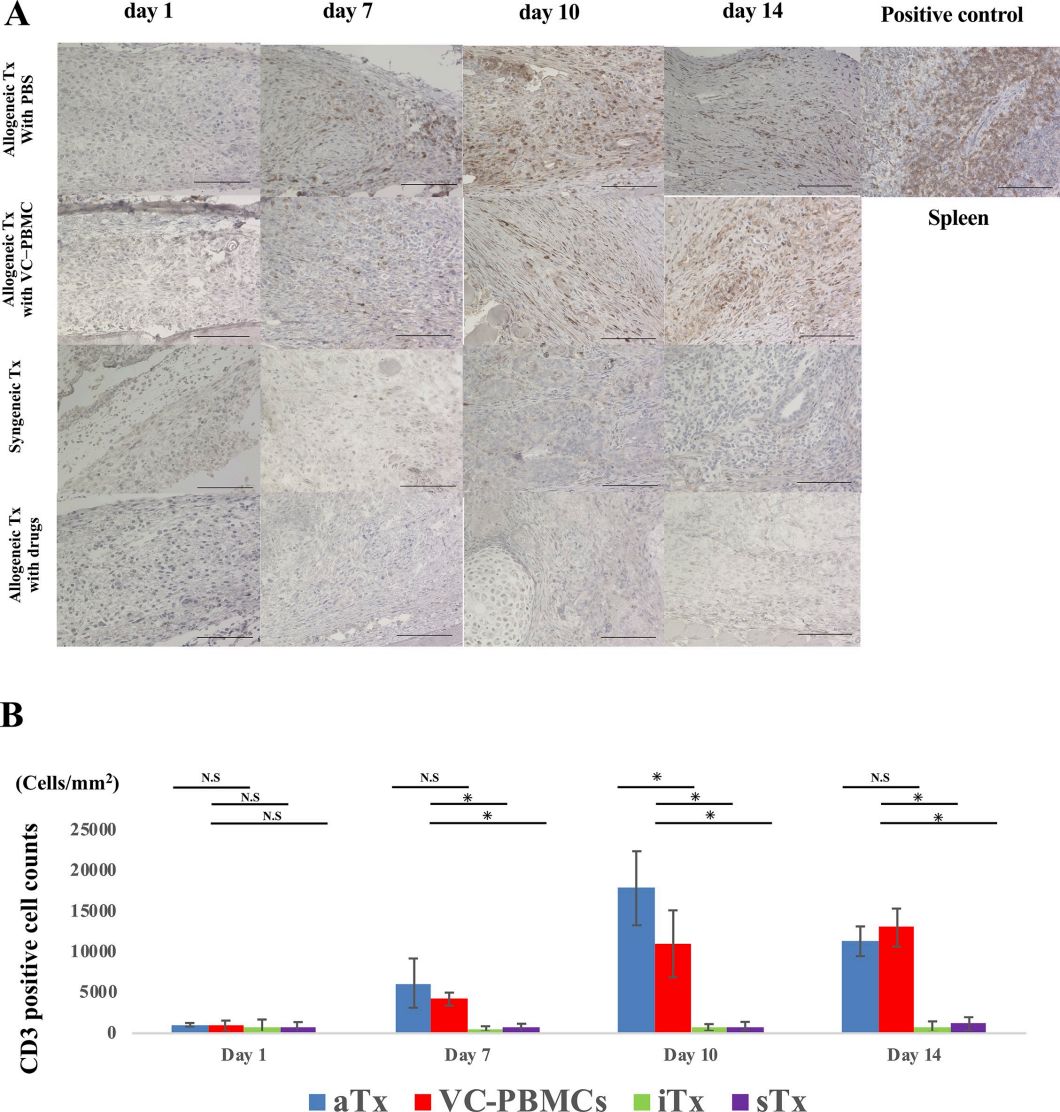
Infiltration of activated T cell lymphocytes into the grafts. **A**, CD3^+^ activated T lymphocytes into grafts at days 1, 7, 10, and 14, using spleen tissue as positive control. **B**, Comparison among groups of CD3^+^ cell counts. Scale bar, 100 μm. *, *P* < 0.05; N.S., not significant.

**Fig 4:**
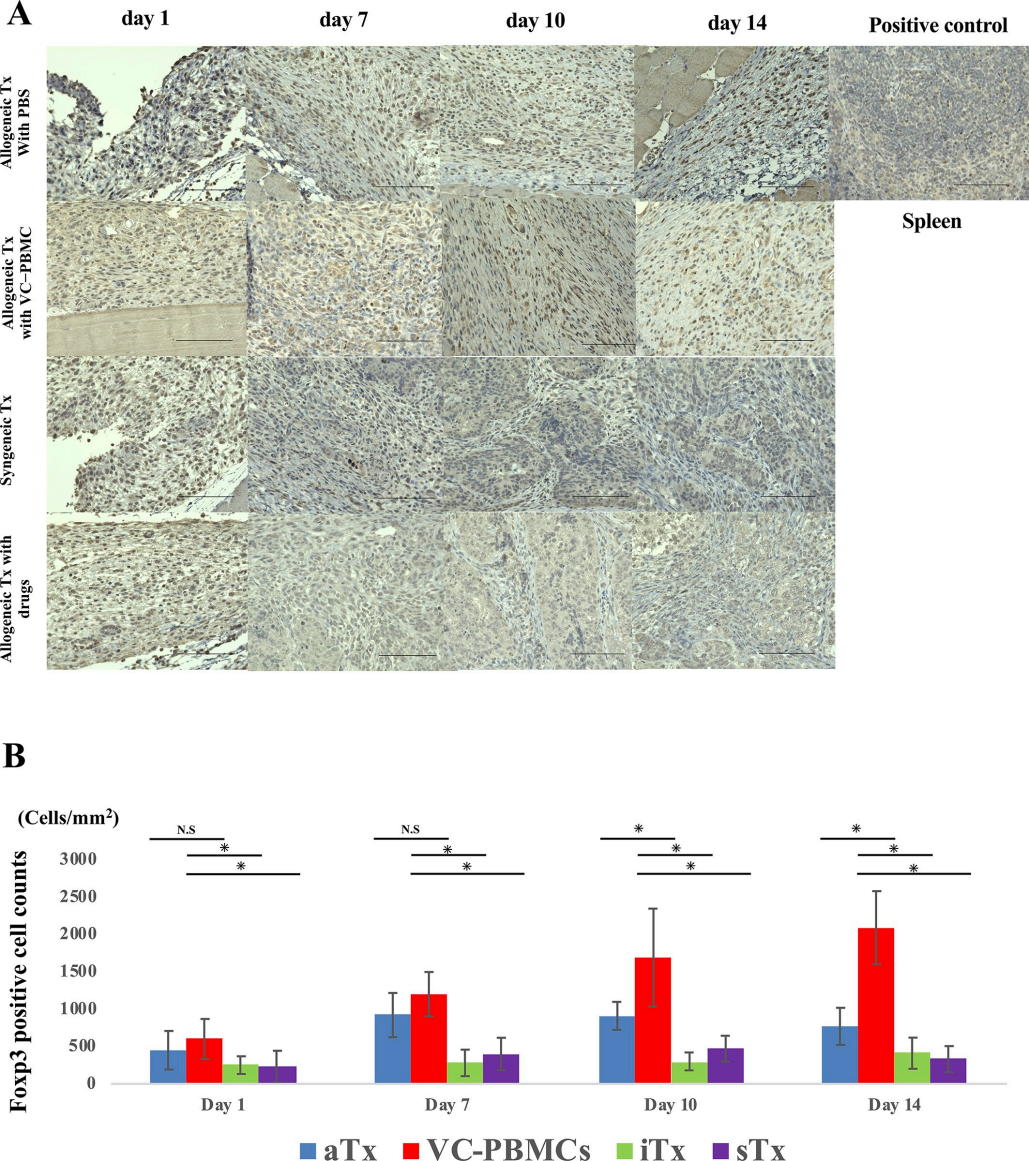
Infiltration of regulatory T cell lymphocytes into the grafts. **A**, Foxp3^+^ regulatory T cells into grafts at days 1, 7, 10, and 14, using spleen tissue as positive control. **B**, Comparison among groups of CD3^+^ cell counts. Scale bar, 100 μm. *, *P* < 0.05; N.S., not significant.

By staining with hematoxylin and eosin, transplanted iPSC-derived cardiomyocyte sheets were observed in subcutaneous tissues collected over time, especially in syngeneically transplanted mice and in allogeneically transplanted mice treated with immunosuppressants. CD3^+^ and forkhead box p3 (Foxp3)^+^ T lymphocytes were not detected in syngeneically transplanted mice and in allogeneically transplanted mice treated with immunosuppressants. The grafts gradually proliferated and formed teratomas, but not in allogeneically transplanted mice injected with or without vasculogenically conditioned peripheral blood mononuclear cells (Fig 2C). CD3^+^ T infiltration was detected in allogeneically transplanted mice injected with or without vasculogenically conditioned peripheral blood mononuclear cells, but was more extensive at day 10 in the latter than in the former (Fig 3A and 3B). Furthermore, Foxp3^+^ regulatory T cells also accumulated in the former, but fewer such cells were observed in the latter, especially at day10 and 14 (Fig 4A and 4B).

### Gene expression at graft sites

**Fig 5:**
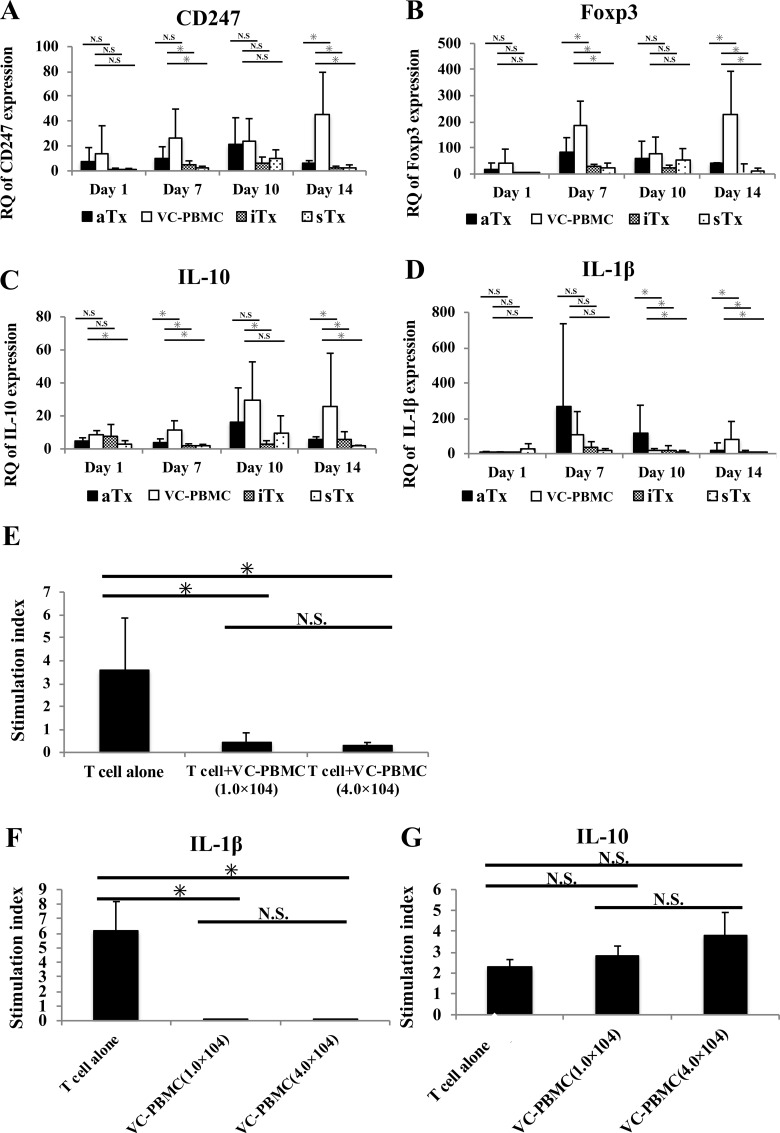
Expression of immune markers at transplant sites. **A-D**, Time course of CD247, Foxp3, IL-10, and IL-1γ expression after transplantation; **E**, T cell proliferation (4.0 × 10^4^ cells) in the presence or absence of vasculogenically conditioned peripheral blood mononuclear cells (1.0 × 10^4^). **F-G**, Release of immune cytokines, such as IL-10 and IL-1γ, from T cells (1.0 × 10^4^) stimulated with CD3 and CD28 in the presence or absence of vasculogenically conditioned peripheral blood mononuclear cells (4.0 × 10^4^ cells). *, *P* < 0.05; N.S., not significant.

CD247, a marker of T cells, was abundantly expressed in subcutaneous tissues from allogeneically transplanted mice treated with or without vasculogenically conditioned peripheral blood mononuclear cells, but significantly more abundant in the former than in the latter (Fig 5A). Foxp3, a marker of regulatory T cells, was also significantly more abundant at day 14 in allogeneically transplanted mice treated with conditioned cells than in any other treatment group (Fig 5B). Importantly, IL-10, an immunosuppressor, was most strongly expressed at day 14 in allogeneically transplanted mice treated with conditioned cells (Fig 5C), although IL-1β, a marker of active inflammation, was more strongly expressed at day 7 and 10 in allogeneically transplanted mice than in other treatment groups (Fig 5D). CD68, a marker of macrophages, was abundantly expressed especially at day 14 in allogeneically transplanted mice treated with conditioned cells. Interestingly, CCR2 and NOS2, which are markers of activated M1 macrophages, and mannose receptor and arginase-1, which are markers of regulatory M2 macrophages, were also strongly expressed in these animals throughout the experiment. Levels of other cytokines, including IL-2, IFN-γ, and TNF-α, are plotted in Supporting Information 1A-H.

### Vasculogenically conditioned cells suppressed T cell proliferation with suppressing IL-1β and stimulating IL-10 secretion

T cells significantly proliferated upon stimulation by CD3 and CD28 antibodies, but not in the presence of vasculogenically conditioned peripheral blood mononuclear cells, although the suppressive effect did not appear to be dose-dependent (Fig 4E). IL-1β secretion also diminished in the presence of conditioned cells, confirming that the T cells are suppressed (*P* < 0.01). On the other hand, IL-10 secretion was modestly stimulated in the presence of conditioned cells (*P* = 0.1, Fig 4F and 4G).

## Discussion

Although allogeneic iPSCs are progressing toward clinical use in cardiac regenerative therapy because of significant time and cost savings in comparison to autologous iPSCs, the ensuing immune reaction in the recipient remains an issue [5–7]. Accordingly, strategies to minimize immune activity against allogeneic cell transplants have been considered, including immunosuppressive agents [17], major histocompatibility complex (MHC) matching against an MHC-homo iPSC bank [3], and cotransplantation with immunosuppressive cells [18]. We note that although immunosuppressive drugs are already used routinely in solid organ transplantation [19], their value in cell transplantation is still unclear. Furthermore, side effects from such drugs, including susceptibility to infection, malignancy, and renal dysfunction, are not completely avoidable [20]. On the other hand, MHC-matched transplantation has no side effects, but was shown to be effective in allogeneic iPSC transplantation into various organs such as the brain, eye, and heart [3, 6, 21]. Nevertheless, even MHC homo-to-hetero transplantation may still elicit immune rejection via natural killer cells, ligand mismatch to killer cell immunoglobulin-like receptors [8], or allorecognition against minor antigens [9]. Thus, further investigation is required to establish suitable immunological protocols following iPSC transplant.

In this study, we investigated the possibility that vasculogenically conditioned peripheral blood mononuclear cells may suppress host immunity against allogeneic iPSC-derived cardiomyocyte sheets. We note that vasculogenic conditioning was originally intended to expand endothelial progenitor cells, and to obtain myeloid cells with vasculogenic potential. Following conditioning *in vitro*, regulatory T cells accumulated, and some immunosuppressive cytokines such as IL-2 and IL-10 were released to the culture medium. Conditioned cells also stimulated IL-10 secretion from T cells *in vitro*, but suppressed proliferation and secretion of IL-1β, a marker of acute inflammatory activation. In subcutaneous tissue from mice transplanted with iPSC-derived cardiomyocytes and injected with conditioned cells, IL-10 and Foxp3, a marker of regulatory T cells, were abundantly expressed, while IL-1β was only modestly expressed. Taken together, the data suggest that vasculogenically conditioned peripheral blood mononuclear cells are immunosuppressive, and mitigate the immune response against donor antigens via regulatory T cells, in line with similar studies. For example, Sasaki et al [18]. reported that differentiated macrophage-like suppressor cells from the same source as the iPSC-derived graft prolong its survival primarily via iNOS-mediated suppression of host T- and B-cells. Similarly, we observed that vasculogenically conditioned cells significantly extended graft survival. Interestingly, graft survival was not significantly different between allogeneic transplantation with and without conditioned cells at the end of this study. We speculated that this is because the effect of conditioned cells to suppress host immune reaction may not be enough to resuscitate the allogeneically transplanted grafts for a long duration, because syngeneic grafts and allogeneic grafts with immunosuppressant showed tumorigenic proliferation over time by avoiding host immune response. Therefore, further investigation should be needed for the future study about how conditioned cells works after being injected and what would be the best way to deliver the conditioned cells to resuscitate the allogeneic grafts for a longer duration.

Vasculogenically conditioned peripheral blood mononuclear cells are enriched not only in endothelial progenitor cells and anti-inflammatory M2 macrophages, but also immunosuppressive lymphocytes (Th2 regulatory T cells). In contrast, inflammatory M1 macrophages and immunogenic lymphocytes (B-lymphocytes, natural killer cells, and cytotoxic T cells) are not as abundant [12, 13]. Thus, vasculogenically conditioned cells represent a polarized population of peripheral blood cells primed for vascularization, anti-inflammation, and immune tolerance, and in this sense resemble regenerative cells associated with ischemia or tumors [14]. Indeed, regulatory T cells are strongly associated with immune tolerance following allogeneic cell transplantation [22], and were shown to prevent acute graft-versus-host disease after allogeneic bone marrow transplantation [23], and to prolong allograft survival after renal transplantation [11]. Some mechanisms of tolerance induced by regulatory T cells include deletion of thymus or peripheral T cells that react to donor alloantigens [24], T cell anergy [25], and desensitization to alloantigens. IL-10 is also well-known to induce and maintain tolerance [26, 27], while IL-2 activates and maintains regulatory T cells [28]. Hence, the immunosuppressive properties of vasculogenically conditioned peripheral blood mononuclear cells are probably due to IL-2 and IL-10 release, as observed *in vitro*, or to IL-10 release and suppression of IL-1β, as observed in the graft.

Although generating regulatory T cells *ex vivo* may appear to be a more straightforward approach to minimize immune rejection, it is unclear whether regulatory T cells alone are effective, since mechanisms that induce immune tolerance are complex and depend on an array of cells and cytokines. Therefore, a heterogeneous pool of cells, such as vasculogenically conditioned peripheral blood mononuclear cells, may be more effective. Indeed, these cells also include regulatory macrophages that strongly express CD68, arginase-1, and NOS2 [15], which may reinforce immune suppression. In addition, strongly vasculogenic endothelial progenitor cells that are enriched after conditioning and express CD34 may enhance paracrine effects from grafts, although further investigation is needed to investigate this possibility. Most importantly, vasculogenically conditioned cells can be quickly established from the recipient’s peripheral blood without invasive bone marrow aspiration and without complicated cell purification, suggesting that this strategy could be widely usable in light of cost and time advantages.

It is possible that results from a single line of iPSCs, in this case 959A2-1, are insufficient to draw valid conclusions, although this cell line is well characterized, and is consistently differentiated into cardiomyocytes. In addition, further studies using human iPSCs transplanted into large animals may be needed as preclinical proof-of-concept. Using healthy mice and specific transplantation sites are additional limitations of the study, because morbidities and other transplantation sites may generate dissimilar immune reactions. Accordingly, further studies using a murine model of infarcted heart or clinical heart failure would be needed to definitively demonstrate the effectiveness of cotherapy with allogeneic iPSCs and vasculogenically conditioned peripheral blood mononuclear cells. Finally, while the immunosuppressive effects of conditioned cells are not particularly robust, they may nevertheless lower the required dose of immunosuppressive agents, and thereby help minimize potential side effects.

## Conclusions

Concomitant transplantation of vasculogenically conditioned peripheral blood mononuclear cells prolongs the survival of subcutaneously grafted allogeneic iPSC-derived cardiomyocytes *via* regulatory T cell-mediated inhibition of host immunity. Thus, we anticipate that cell-based immunosuppression may enhance clinical outcomes.

## Supporting information

S1 Fig Time course of gene expression of markers of inflammation and immune reaction. **A-H**, CD68, arginase-1, mannose receptor (MARC), NOS2, chemokine receptor 2 (CCR2), interleukin-2 (IL-2), interferon-β (IFN-β), and tumor necrosis factor-α (TNF-α) after transplantation.
(TIFF)
